# Gene Disruption Technologies Have the Potential to Transform Stored Product Insect Pest Control

**DOI:** 10.3390/insects7030046

**Published:** 2016-09-19

**Authors:** Lindsey C. Perkin, Sherry L. Adrianos, Brenda Oppert

**Affiliations:** Center for Grain and Animal Health Research, Agricultural Research Service, USDA, 1515 College Avenue, Manhattan, KS 66502, USA; Lindsey.perkin@ars.usda.gov (L.C.P.); Sherry.adrianos@ars.usda.gov (S.L.A.)

**Keywords:** stored product pests, pest management, RNAi, CRISPR

## Abstract

Stored product insects feed on grains and processed commodities manufactured from grain post-harvest, reducing the nutritional value and contaminating food. Currently, the main defense against stored product insect pests is the pesticide fumigant phosphine. Phosphine is highly toxic to all animals, but is the most effective and economical control method, and thus is used extensively worldwide. However, many insect populations have become resistant to phosphine, in some cases to very high levels. New, environmentally benign and more effective control strategies are needed for stored product pests. RNA interference (RNAi) may overcome pesticide resistance by targeting the expression of genes that contribute to resistance in insects. Most data on RNAi in stored product insects is from the coleopteran genetic model, *Tribolium castaneum*, since it has a strong RNAi response via injection of double stranded RNA (dsRNA) in any life stage. Additionally, Clustered Regularly Interspaced Short Palindromic Repeats (CRISPR) technology has been suggested as a potential resource for new pest control strategies. In this review we discuss background information on both gene disruption technologies and summarize the advances made in terms of molecular pest management in stored product insects, mainly *T. castaneum*, as well as complications and future needs.

## 1. Introduction

New gene disruption technologies, such as RNA interference (RNAi) and Clustered Regularly Interspaced Palindromic Repeats (CRISPR) are rapidly expanding in many facets of biological research. These gene disruption tools have proved useful for a number of applications, including disease models [1], gene mapping [2], and functional genetics [3,4,5]. Agriculture researchers also are developing ways to apply these technologies to farming and food storage needs, as well as integrated pest management (IPM) [6,7,8].

Stored product insect species adversely affect grain in storage, milling facilities, warehouses, and even the consumer pantry [9]. The economic impact of grain production in the United States alone is estimated at $115 billion annually, and as grains are processed and manufactured into human and animal food products, the value increases dramatically. The United States food manufacturing industry segments that are impacted by stored product insects are valued at over $300 trillion annually. Insects destroy an estimated 5%–10% of stored grain in developed countries and >30% in developing countries. The exact costs of stored product insects inflicted on the food industry is difficult to precisely measure, but represents a significant impact due to losses contributable to food contamination and costs associated with prevention and treatment activities.

Traditional treatments for stored product pests have tended to rely on fumigation using phosphine (PH_3_) and methyl bromide (MeBr), but use of IPM strategies to reduce the need to fumigate is increasingly important. MeBr usage as a fumigant is being phased out worldwide, except for quarantine and pre-shipment treatments, because it is an ozone-reducing chemical. Insect populations around the globe are rapidly developing resistance to PH_3_, the most commonly used fumigant for bulk-stored grain, and in some cases to very high levels [10,11,12,13]. Use of reduced risk insecticides, such as pyrethrins and insect growth regulators, are increasing as alternatives to fumigation, but these insecticides also have their limitations [14,15,16]. As an alternative to fumigation, molecular-based treatments promise target specificity and less damage to the environment.

Much of the available literature on gene disruption in the agricultural field has focused on the red flour beetle, *Tribolium castaneum*, the current genetic model for coleopteran agricultural pests [17]. *T. castaneum* is a pest in milling facilities, and is an excellent model for molecular-based pest control studies because of the genetic information available. Arguments for using this species as a model for genetic technology are: relatively quick generation time and easy to rear in the lab; resistant strains (Table 1) [18,19] and mutants are maintained and available for public dissemination (http://spiru.cgahr.ksu.edu/proj/tribolium/region.asp); all life stages have an inducible response to RNAi; there is a developing RNAi database (iBeetle) [20]; and success with CRISPR has been documented [21]. Much of what we have learned from *T. castaneum* may be applicable in other stored product pests, but currently the main hurdle is the limited genomic information available. Therefore, in this review, we will focus on *T. castaneum*, and the advances made for potential agricultural use of two gene disruption technologies, RNAi and CRISPR. We will describe the background and mode-of-action of each technology, the progress of each in *T. castaneum*, and speculate on new ideas for application, as well as problems and recommended areas of further study.

## 2. Technology Background

### 2.1. RNAi

In nature, RNAi initiates when long double stranded RNA (dsRNA) is introduced into an organism via infection. Once the dsRNA is introduced, the endoribonuclease *Dicer* cleaves the dsRNA into 21–23 nucleotide fragments, which are referred to as short interfering RNA (siRNA). The unwound single-stranded guide strand of the siRNA is incorporated into an *RNAi-induced silencing complex* (*RISC*) that targets and degrades RNA with complementary sequence. The first discovery in animals was in the nematode, *Caenorhabditis elegans* [23], whereby the induced dsRNA moves from cell to cell throughout the entire body via a systemic response. There are many demonstrated methods to administer dsRNA, such as injecting, soaking, and feeding. In contrast to *C. elegans*, RNAi has not been fully characterized in insects. Tomoyasu et al. [24] searched for RNAi machinery orthologs in *T. castaneum* and found the core components, *Dicer*, *RISC*, *Argonaute*, SID-1 orthologs. A more complete review of RNAi machinery in insects can also be found in Huvenne and Smagghe [7] and Scott et al. [25].

Achieving successful RNA knockdown is dependent on many factors, including length and concentration of the dsRNA fragment, nucleotide sequence specificity, life stage of the organism, and genetic background [7,26]. In *T. castaneum*, directly injecting dsRNA into any life stage can result in gene silencing [27,28]. Miller et al. [29] found that dsRNA fragments of about 520 base pairs were sufficient to knockdown target gene expression in larvae at concentrations ranging from 0.1 to 1.0 ng/μL. In *T. castaneum*, the maternal genetic background can influence the RNAi phenotype, but does not change overall RNAi sensitivity [26]. Thus, care should be taken when choosing an experimental strain, and injecting multiple strains may uncover genetic diversity.

Functional-based RNAi studies were first done in *T. castaneum* by Brown et al. [5] to demonstrate homology in developmental pathways of *T. castaneum* and *Drosophila melanogaster*. Many successful RNAi experiments followed, identifying genes that cause developmental abnormalities and/or mortality, and thus are potential candidates for pest control genes. Several studies have focused on chitin and cuticle development. For example, Arakane et al. [30] analyzed the function of the *chitin deacetylase* (*CDA*) gene family, identifying tissue specific expression and the effects of RNAi targeting one or multiple *CDA* genes. Of the nine *CDA* genes, half were expressed in the larval gut (*CDA6-9*), and the other half (*CDA1-5*) expressed in epidermal and/or tracheal cells. RNAi knockdown of *CDA2a* and/or *CDA2b* in adult females resulted in F1 progeny with molting issues, unable to either hatch or progress to second larval instar (approximately one day post hatch). Additionally, RNAi of two chitin synthase genes (*CHS1* and *CHS2*) revealed each had a unique function [31]. The data indicated that *CHS1* was involved in whole-body chitin content, and knockdown disrupted all types of molting (larval-larval, larval-pupal, and pupal-adult). RNAi of *CHS2* led to smaller larval size, reduced chitin in the midgut, and a cessation of larval feeding.

Cuticle development (sclerotization and pigmentation) in *T. castaneum* is a complicated process involving many different genes. Some cuticular genes are involved in coloration, and others are part of the structural cross-linking for cuticle integrity. Knockdown of the *aspartate 1-decarboxylase (ADC)* gene resulted in a black cuticle instead of the wildtype red-brown, but also reduced the overall cuticle strength, with *ADC* mutants having reduced cross-linking in their cuticle [32]. Interestingly, *ADC* is located on chromosome 3, and mutations of *T. castaneum* with black cuticle phenotype have been mapped to linkage group 3 (Table 2); it is unknown if these mutants have *ADC* deficiencies, or other changes in cuticular genes. Similarly, RNAi knockdown of the *yellow-e* gene in larvae caused adults to have a yellowed, crumpled dorsal cuticle and to be intolerant to low relative humidity (RH), resulting in mortality at levels less than 100% RH [33]. Thus, the reduction in *yellow-e* gene expression reduced the waterproofing characteristics of the cuticle.

In addition to small-scale functional tests, the group iBeetle (http://ibeetle-base.uni-goettingen.de) has conducted a large-scale, genome-wide RNAi screen [20,34]. The iBeetle screen involved injecting both female pupae and 5th/6th instar larvae with dsRNA targeted to approximately one-third of the total genes in the *T. castaneum* genome, and each injected insect was scored for morphological phenotypes and sterility. The offspring that resulted from the pupal injections were also scored for phenotype and percent hatch [20]. This database provides a wealth of information on gene functions and is searchable for specific genes. Using this resource, Ulrich et al. [34] scanned for genes that led to the highest and fastest mortality when disrupted. They reported the top 11 genes, with products mostly associated with the proteasome to be the most effective RNAi targets, all of which resulted in 80%–100% mortality by eight days post injection.

Of course we discuss only a small subset of RNAi studies here. There are many more studies that reveal target genes or pathways with potential in disrupting reproduction [35], molting [36], biosynthesis of juvenile hormones [37], digestion [38], metabolism [39], and immunity [40], among others.

### 2.2. CRISPR

**Figure 1:**
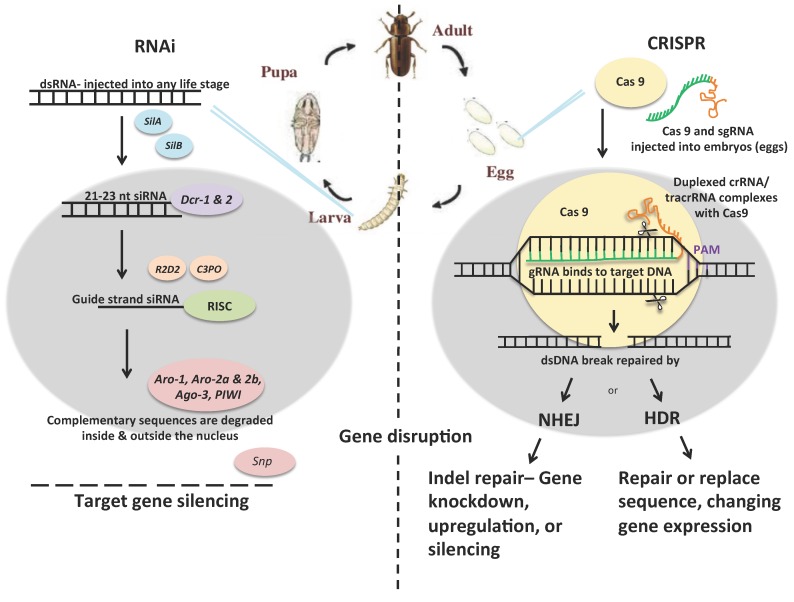
Schematic of RNA interference (RNAi) (**a**) and CRISPR-Cas (**b**) technology in *Tribolium castanem*. The grey oval represents the cell nucleus. On the RNAi side (**a**), dsRNA can be injected into any life stage. The double stranded RNA (dsRNA) is incorporated into the cell with *SilA* and *SilB* (SID-1 orthologs). *Dcr-1* and *2* (*Dicer*) cleave the dsRNA into 21 nt pieces, *R2D2* and *C3PO* help load the RNAs into the silencing complex. *Aro-1, 2a, 2b, 3,* and *PIWI* endonucleases degrade the complementary RNA inside the nucleus while *Snip* (*SNP*) exonuclease degrades the complementary RNA outside of the nucleus. On the CRISPR side (**b**), Cas9 and single guide RNA (sgRNA) is injected into eggs. Duplexed crRNA/tracrRNA complexes with Cas9 endonuclease, resulting in a precise dsDNA break. The break is repaired by either non-homologous end joining (NHEJ) or homology-directed repair (HDR), which results in gene knockdown, upregulation, silencing, or changes in gene expression.

CRISPR and the endonuclease CRISPR-associated protein 9 (Cas9) system (CRISPR-Cas9) originated from the innate immune system of bacteria. Unlike RNAi, which disrupts gene expression, CRISPR-Cas9 is a powerful DNA editing technology that not only disrupts gene expression, but also alters or even inserts coding sequences. In bacteria, foreign DNA sequences integrated into DNA are targeted by the CRISPR-Cas9 system as part of a defense mechanism that enables bacteria to ward off infections from viruses and bacteria [41]. CRISPR-Cas9 uses a guide RNA (gRNA) to find and form base pairs with a DNA target sequence, and binds the Cas9 endonuclease which cuts the double stranded DNA (dsDNA) very precisely (Figure 1b) [42,43,44]. The DNA break is repaired by non-homologous end joining (NHEJ) or homology-directed repair (HDR) by endogenous cellular machinery. When dsDNA breaks are repaired by NHEJ, a single or multiple nucleotide insertion or deletion (INDELS) often occurs, and can shift the reading frame of the gene sequence, effectively turning the gene off. When no INDELS occur, the DNA is returned to its original state and no change occurs. HDR requires the incorporation of an additional template component containing the desired altered sequence, flanked by sequences homologous to either side of the cut site. In HDR, homologous recombination is utilized to incorporate new sequences to repair or introduce genes.

CRISPR genome editing is simpler, more cost effective, faster, and easier to use than previous genome editing technologies, like transcription activator-like effector nucleases (TALEN) and zinc finger nucleases, and facilitates precise and efficient targeting, editing, modification, and regulation of cells and organisms [45]. Off-target effects are less of an issue as a better understanding of CRISPR is emerging. Different caveats of the various system components have led to optimized methods that reduce or eliminate off-target effects [46].

CRISPR was first proposed as a simplified genomic editing tool in 2012 [43]. Adaptations for use in eukaryotic cells were reported in human and mouse trials a short time later [47]. Since 2012, CRISPR-Cas9 has proven to be a powerful research tool for gene editing, genome-wide screens, studying individual gene function, creating disease models, and working towards potential therapeutics for human and animal health [1]. CRISPR technology was ranked as the Science Magazine “Breakthrough of the Year” in 2015 [48] because of the potential application to fields such as agriculture and medicine. In the CRISPR-Cas9 gene editing system for research use, endonucleases (like Cas9 and others) and gRNA have been continuously optimized for accuracy and efficiency [46].

Advancing CRISPR technology holds gene-editing promise for insect model organisms such as *T. castaneum* [49]. A review that includes a discussion on non-model insect genome editing provides advice for transferring gene-editing technology to other insects [50]. CRISPR gene disruption has been demonstrated in insect cell lines [51] and in the embryo or larval/immature stages of insects such as fruit flies, silkworms, mosquitoes, and others [50,52,53,54,55,56]. The first and only report of CRISPR technology utilized in a stored product pest was in *T. castaneum* for gene targeting and transgene replacement [21], with more reports anticipated to follow. The development of a system that utilizes the *T. castaneum* U6 promoter (or other more effective promoters) for expression in plasmid delivery systems will further advance CRISPR studies in *T. castaneum*.

### 2.3. RNAi vs. CRISPR as Insect Control Strategies

CRISPR edits the DNA of the cell, thereby changing gene expression permanently if it is a stable transformation. RNAi interferes with existing gene expression and has diminishing effects unless dsRNA is continuously administered, although in some cases a parental RNAi effect has been documented [57]. A modification of the RNAi technology called CRISPR interference (CRISPRi) has reversible effects, but targets DNA instead of RNA. CRISPRi uses a catalytically deactivated Cas9 (dCas9) that reversibly binds to target DNA to inhibit gene expression [58,59]. CRISPR does not interfere with the endogenous cellular machinery, which can be a problem with siRNAs or short hairpin RNAs (shRNA) that may cause cell death [45]. While there are advantages of CRISPR for permanent gene modification, RNAi has advantages in applied use, whereas CRISPR technology has thus far been limited by delivery methods as well as biosafety containment considerations. RNAi and CRISPR gene disruption technologies complement each other in gene function research.

## 3. Application in Pest Management

### 3.1. Current Delivery Mechanisms

Implementation and delivery mechanisms present unique advantages and challenges for stored product pests. Unlike field pests that are feeding on actively growing plants, grain and grain products are post-harvest and destined for human or animal consumption. Grain is typically treated and stored in large storage structures such as large bins, silos, and bunkers, where it must stay dry, clean, and uninfested. Current stored product pest management of bulk grain relies mostly on fumigation and aeration to lower grain temperature. Food processing facilities are large indoor facilities where insects are found in hidden and hard to reach areas, and the facility must stay clean and meet government and industry standards for human consumption. Facilities for processed grain focus on prevention of infestation by exclusion of insects through sanitation, treating surfaces like concrete, treating cracks and crevices where food material can accumulate, and using insect resistant packaging [9]. New molecular techniques will need to be adaptable to these diverse situations.

Some delivery methods that might be compatible with molecular techniques include grain protectants, surface or crack and crevice treatments, and lure and kill products, each with specific applications. Grain protectants are residual insecticides that are applied to the grain as it is being transferred to a storage bin, and can be applied to all of the grain or just to the top layer of grain in a bin [9]. The main issue with developing molecular products to incorporate into grain protectants is the cost, as well as limited types of protectants and efficacy against storage pests. Insecticides can also be applied to surfaces or cracks and crevices in empty bins, equipment and structures where food material accumulates, and it’s conceivable that dsRNA could be used in these products. Lure and kill methods involve attractants to draw insects to a location where they are exposed to an insecticide, another possibility for dsRNA. Lure and kill requires less insecticide and reduces the hands-on time of identifying and treating all infested locations with insects, so it has more potential for molecular products [60].

Attractants used for both monitoring traps and lure and kill can be chemical cues such as pheromones or kairomones or visual cues such as specific wavelengths of light [61,62,63]. Pheromones are often species and sex specific, which can be an advantage if targeting a specific pest, but pheromones are commercially available for only some of the common pest species, and not all species respond strongly. *T. castaneum* has an aggregation pheromone that attracts males and females, but attraction is limited under conditions that lack air movement [62]. Many commercially available pheromones are sex pheromones that attract males, which can successfully impact population growth. For example, Entostat (Exosect, Hampshire, UK) is a pheromone-laden powder that attracts males that transfer the powder to other males and females through contact and thus propagate through a population [64,65]. Kairomones, or food odors, can attract a wider range of species and both sexes, but need to compete against other food odors in the environment [62]. Sequenced genomes and transcriptomes from stored product insects are providing data on sensory genes that will lead to the development of better lures [66,67].

### 3.2. New Delivery Mechanisms

One of the major issues in using RNAi and CRISPR as a stored product pest control strategy will be administering it to insects on a large scale. The opportunities to use gene disruption in stored product pest management are limited by economics and background research. CRISPR technology in *T. castaneum* needs further development for pest control applications. RNAi in *T. castaneum* is reproducible and reliable, but to date is only routinely successful via introduction by micro-injection, a process that is not possible on a large scale. Feeding, which has not been widely successful, or topical application, which has not been studied, would be better alternatives. One study found that dsRNA targeting *vATPase* and fed to *T. castaneum* as part of their diet elicited a response [68], but replication of this study by others has not yet been documented. Unfortunately, the majority of applications for RNAi in the field rely on oral delivery. A recent report indicated that a transgenic bacterial symbiont was used to colonize the gut and successfully block a key fecundity gene in a major Chagas disease vector, *Rhodnius prolixus*, resulting in significantly less offspring [69]. As some stored product insects contain bacterial symbionts [70,71,72], this provides an exciting area to explore.

Successful oral RNAi has been achieved through transgenic maize targeting field pests, such as the western corn rootworm [6,73]. RNAi also has been applied to other cereal crops, such as wheat and barley [74], and CRISPR has been demonstrated in rice and wheat [75], but mostly these crops have been modified for nutritional value and yield. Modification of the seed kernel for protection against insect pests has been avoided due to regulatory concerns. In maize genetically modified to express dsRNA targeting the gene *Snf7* for rootworm control, the transgene is expressed at low levels in the kernel (0.1 ng/g grain), but expression is much higher in plant tissue where the insect damage occurs (up to 55 ng/g) [76]. However, early in the production of transgenic maize, insecticidal proteins from *Bacillus thuringiensis* expressed in the kernel provided protection against lepidopteran storage pests and was proposed as a management tactic for maize pests in bins [77].

At some point, regulatory concerns about the expression of dsRNA in grain may diminish, as more studies are available on the impact of human and animal health. However, as we gain information on cereal and insect genomes through sequencing and gene disruption studies, it may be possible to identify and modify cereal genes to increase the production of innate proteins with a negative impact on storage pests, a kind of “accelerated evolution”. This approach would improve the control of pests in grain and stored products without inclusion of foreign genes in transgenic cereals, and thus diminish regulatory concerns. This type of technology has been pursued in the tomato, where naturally produced chemicals, like jasmonic acid, detour herbivorous insects [78].

Another problem is the cost of producing dsRNA. Limited amounts of dsRNA can be made in the laboratory using commercial kits, such as MEGAscript (Thermo Fisher Scientific, Waltham, MA, USA). Most RNAi experiments have used this method to manufacture and test the effects of dsRNA by injection. However, feeding experiments require much larger quantities than can be obtained through in vitro methods. Bacteria can be used to serve as a factory for producing larger quantities of dsRNA in vivo. Engineering bacteria to express dsRNA requires the use of RNase III defective bacteria. The HT115 (DE3) *Escherichia coli* strain and pL4440 plasmid, developed for dsRNA delivery in *C. elegans*, is the most widely used system [23]. The pL4440 plasmid was designed with two T7 promoters (double T7) and the gene of interest placed between them, resulting in the transcription of dsRNA in HT115 cells transformed with the transgene-containing plasmid. More information on this system and kit requests can be found through the Carnegie Institution of Washington (www.ciwemb.edu) or Addgene (www.addgene.org). This system has been demonstrated to work in the coleopteran, *Leptinotarsa decemlineata* (Colorado potato beetle), where suppression of the target gene reduced larval feeding and induced mortality when beetles were fed either with dsRNA made in vitro (MEGAscript kit) or in vivo (HT115 (DE3)) and pL4440 [79].

Yeast as a dsRNA delivery method has been reported only recently in *Drosophlia suzukii* [80]. The benefits to using yeast over bacteria are that it is part of a normal *T. castaneum* diet and may be more nutrient-rich than the grain itself, and thus, given a choice, beetles might prefer to eat the recombinant yeast. Certain strains of yeast are already found in the stored product pest environment and pose no threat to humans. The application of bacteria and yeast dsRNA expression methods could be developed into seed protectants or sprays that could be applied in cracks and other locations where food material accumulates, or to treat baits in a lure and kill application.

Methods have been developed to encapsulate nucleic acids into nanoengineered, degradable capsules that can be used in diverse applications [81]. Each capsule is layered with nucleic acid that can be released and used in such applications as polymerase chain reaction (PCR), and is designed with a structural integrity to remain active over time. Putting this technology to practice, Zhang et al. [82] demonstrated that dsRNA-based nanoparticles could inhibit gene expression in the mosquito *Anopheles gambiae* larvae. To our knowledge, nanoparticles have not been studied for use in stored product pest control, but doing so could provide new application options in IPM. Encapsulated dsRNA beads could be engineered and added to bulk grain, as well as placed strategically in grain processing facilities, in the hidden areas where insects feed and develop, or as part of a top layer for grain in storage. These nanoparticles offer the possibility of a biodegradable, food safe method of stored product pest control.

Utilizing CRISPR for engineered gene drives to manipulate organisms has the potential to reverse the development of resistance to insecticides in stored product pests [83]. Gene drives function to “drive” a gene of interest into or out of a wild population. Gene drives have been designed for organisms such as mosquitoes, fruit flies, and yeasts [84,85,86]. CRISPR-Cas9 facilitated gene drive methods have been established in *Drosophila* [87,88]. CRISPR mediated gene disruption facilitated the reduction of pyrethroid pesticide resistance in mosquitoes and demonstrated a dramatic, more than 100-fold reduction in resistance in one haplotype [89]. This method of gene disruption allows for targeting the gene, promoters, and regulatory elements that drive overexpression and are often responsible for resistance. However, genes with extensive copy numbers, such as detoxifying enzymes often responsible for insecticide resistance, are more challenging to disrupt to a level that affects phenotype. The primary concern is that safeguards need to be developed for reverse gene drives to control populations if problems arise with releases of genetically-engineered insects in the wild, a necessary step prior to regulators granting approval. However, given that one of the major resistance genes for PH_3_ (dihidrolipoamide dehydrogenase) has been identified in a number of stored product pests, including *T. castaneum* [90], it is conceivable that stored product pests engineered with copies of the non-mutated gene could be incorporated into a release program to drive PH_3_ resistance genes out of a population.

In addition to resistance genes, we propose other potential targets for CRISPR or RNAi. For example, targeting pheromone and receptor genes by dsRNA or CRISPR could induce incompatibility between male and female cues or reduction in finding a mate. The insect gut may also be a candidate target, such that efficient digestion is compromised. Stored product beetles, such as *T. castaneum* and *Tenebrio molitor*, primarily use cysteine peptidases to digest food [91,92,93], and targeting genes that encode specific cysteine peptidases or other critical digestive peptidases by dsRNA or CRISPR may slow growth or impede development. Driving immune-deficient genes throughout the pest population via CRISPR gene editing could reduce population levels by eliminating the ability to fight infection or increase susceptibility to insecticidal toxins, such as those produced by *B. thuringiensis*.

## 4. Complications

Like all new technologies, RNAi and CRISPR come with potential complications and limitations. In this section we highlight some of the main issues in terms of developing environmentally friendly, logistically possible, and inexpensive pest management tools.

### 4.1. RNAi

One of the most discussed issues with RNAi in pest management is the problem of off-target effects and contrasting opportunity for pest specificity. Ulrich et al. [34] demonstrated that many candidate target genes thus far have orthologs in other insects. It will be essential that all candidate genes are rigorously tested and carefully selected in order to eliminate the possibility of off-target effects in other insects, especially beneficial insects, such as honeybees. One advantage for stored products is that treatments are inside facilities and grain bins, reducing the opportunity for off-target species contact with the treatment. As more genomes are sequenced, including stored product insects and non-targets, the ability to identify species-specific sequences will be enhanced through bioinformatic screening.

Other limiting issues are those dealing with the integrity and potency of the dsRNA itself. Concerns include the length of time dsRNA remains active in the environment: too long could be harmful to a beneficial insect, and not long enough would reduce potency. Research is not yet available on the stability of dsRNA in stored product insects. Endonucleases in *Lygus lineolaris* salivary glands can degrade dsRNA similar to commercial RNase III [94]. More research is needed on the stability of dsRNA in the alimentary tract of stored product insects, as well as in the application environment.

Lastly, there are concerns with the potential for insect populations to become resistant to RNAi. Stored product insects have been successful in developing resistance to many insecticides, including phosphine, malathion, pyrethroids, and deltamethrin [18,19,90]. Will they be able to find a way around RNAi mediated knockdown? Insects may acquire viruses with RNAi suppressors, the RNAi machinery could become overwhelmed with high doses of dsRNA, or genetic variation that is found within insect populations could lead to selection of RNAi-insensitive populations—all scenarios whereby RNAi could be rendered ineffective [25].

### 4.2. CRISPR

CRISPR technology also has potential complications. Two primary issues are off-target effects of gene editing and concern over gene drives. An unintentional off-target effect may be due to sequence similarity between insect species, which was not identified in the bioinformatic studies due to the lack of sequence data, incorrect sequence, and/or single nucleotide polymorphisms (SNPs) which can be found in all insect populations. Most off-target effects are mitigated by careful planning, selecting guides with excellent on-target scores, using a modified endonuclease with dual guides, and modifications to the original CRISPR methods that tailor the gene cassette to the targeted insect’s genetics [46].

There are also concerns that a gene drive system would escape the lab or cause undesired consequences in the wild. This issue is being addressed by new safeguards, designed to “undo” gene editing effects by developing a second gene drive, or conditionally linking to another gene so that expression can be tightly controlled [86,87,95]. Recently, the U.S. Academies of Science gave the go-ahead for gene drive research but cautioned that more research is required before release into wild populations [96]. CRISPR-based gene drive technology in *Tribolium* is currently under investigation in our laboratory and others.

### 4.3. Future Needs

Two primary bottlenecks in developing molecular-based pesticides are the need for sequencing more pest insect genomes and bioinformatics analysis of the mass amount of genetic data that already exist. The cost of sequencing has been decreasing steadily, and in some cases, single-insect sequencing is now possible, eliminating the need for multiple generations of inbreeding. Sequencing data and analysis on emerging pests will help to identify insect-specific vulnerabilities and potentially prevent infestations of new pests, including invasive species.

Stored product insects are easy to rear, with fast generation times for most species. Therefore, we make the argument that stored products insects are a great system to study new, molecular-based pest management strategies. Combining the current molecular data, biological life history and behavior, and engineering technologies will be powerful in determining the next steps to incorporate these strategies into IPM. As with all entomology research, cross discipline collaborations are needed to develop new and creative ways to solve pest problems in safe and environmentally friendly ways. 

## 5. Conclusions

RNAi and CRISPR gene disruption technologies are advancing rapidly. There is a need for research focused on specific pest control and target gene selection in stored product insects. Data on gene functions, as they relate to insecticide resistance, biological insecticides, and natural insect attractants should be a priority. RNAi and CRISPR technologies have created the potential for novel and yet untested stored product pest control methods, gene expression modification, gene editing, and gene modification. Continued discussion among all stakeholders will promote the use of these technologies in appropriate and effective pest management strategies.

## Figures and Tables

**Table 1:** Insecticide-resistant strains of *Tribolium castaneum* (Tc) maintained at the USDA ARS CGAHR (Center for Grain and Animal Health Research) laboratory.

Tc Strain	Resistance	Source
A20 Rdiel	Dieldrin	n/a
QTC-279	Pyrethroid	Collins, P.J. (Australia) [22]
Rdiel BC9 Lab-S	Dieldrin	n/a
BRZ-4, BRZ-5	Organophosphate, Phosphine	Rice patty, Pacheco, (Brazil, September 1987)

**Table 2:** Strains of *T. castaneum* with a black phenotype, all mapped to LG3.

Tc Allele	Name/Alias	Origin
B	Black	Alexander Sokoloff, University of California, Berkeley
B (eve)	Black (eve)	n/a
B (i-2)	Black (i-2)	Jeff Stuart, Purdue
B (New)	Black (New)	n/a
B (ST)	Black (Scott Thomson)	M. Scott Thomson, University of Wisconsin, Riverside
B (t) ^2^	Black (tawny)	C.E. Dyte and Miss Dorothy G. Blackman Ministry of Agriculture, Fisheries and Food, England

^1^ Data from http://spiru.cgahr.ksu.edu/proj/tribolium/region.asp; ^2^ Phenotype of individuals is dark brown.

## Abbreviations

The following abbreviations are used in this manuscript:

RNAi	RNA interference
CRISPR	clustered regularly interspaced palindromic repeats
IPM	Integrated pest management
PH_3_	phosphine
MeBr	methyl bromide
CRISPR-Cas9	clustered regularly interspaced palindromic repeats and associated Cas9 protein
dsRNA	double stranded RNA
siRNA	short interfering RNA
RISC	RNAi-induced silencing complexes
CDA	chitin deacetylase
CHS	chitin synthase
ADC	aspartate 1-decarboxylase
RH	relative humidity
gRNA	guide RNA
sgRNA	single guide RNA
dsDNA	double stranded DNA
NHEJ	non-homologous end joining
HDR	homology-directed repair
INDELS	insertions/deletions
TALEN	transcription activator-like effector nucleases
CRISPRi	clustered regularly interspaced palindromic repeats interference
Cas9	CRISPR associated protein 9 (endonuclease)
dCas9	deactivated Cas9
shRNA	short hairpin RNA
PCR	polymerase chain reaction
SNP	single nucleotide polymorphisms
